# Supplementary material for: Super-silencers are crucial for development and carcinogenesis in B cells
Source: Nat Commun. 2025 Sep 25;16:8395. doi: 10.1038/s41467-025-63329-x (PMC12462470; doi:10.1038/s41467-025-63329-x)
Supplement: Supplementary file 3 — Description of Additional Supplementary Files [file 41467_2025_63329_MOESM3_ESM.pdf]

Description of Additional Supplementary Files

File Name: Supplementary Data 1
Description: SSs and TSs in GM12878

File Name: Supplementary Data 2
Description: SS components with top densities of B-cell-cancer SNVs.

File Name: Supplementary Data 3
Description: Experimental results

File Name: Supplementary Data 4
Description: Cancer samples investigated in this study

File Name: Supplementary Data 5
Description: SSs and TSs in primary B cell
