# Supplementary material for: Super-silencers are crucial for development and carcinogenesis in B cells
Source: Nat Commun. 2025 Sep 25;16:8395. doi: 10.1038/s41467-025-63329-x (PMC12462470; doi:10.1038/s41467-025-63329-x)
Supplement: Supplementary file 9 — Reporting Summary [file 41467_2025_63329_MOESM9_ESM.pdf]

nature portfolio

Corresponding author(s): DAPR NCOMMS-23-40520B

Last updated by author(s): Jul 28, 2025

Reporting Summary

Nature Portfolio wishes to improve the reproducibility of the work that we publish. This form provides structure for consistency and transparency in reporting. For further information on Nature Portfolio policies, see our Editorial Policies and the Editorial Policy Checklist.

Statistics

For all statistical analyses, confirm that the following items are present in the figure legend, table legend, main text, or Methods section.

n/a	Confirmed	
☐	☒	The exact sample size (*n*) for each experimental group/condition, given as a discrete number and unit of measurement
☐	☒	A statement on whether measurements were taken from distinct samples or whether the same sample was measured repeatedly
☐	☒	The statistical test(s) used AND whether they are one- or two-sided<br>*Only common tests should be described solely by name; describe more complex techniques in the Methods section.*
☒	☐	A description of all covariates tested
☐	☒	A description of any assumptions or corrections, such as tests of normality and adjustment for multiple comparisons
☐	☒	A full description of the statistical parameters including central tendency (e.g. means) or other basic estimates (e.g. regression coefficient) AND variation (e.g. standard deviation) or associated estimates of uncertainty (e.g. confidence intervals)
☐	☒	For null hypothesis testing, the test statistic (e.g. *F*, *t*, *r*) with confidence intervals, effect sizes, degrees of freedom and *P* value noted<br>*Give P values as exact values whenever suitable.*
☒	☐	For Bayesian analysis, information on the choice of priors and Markov chain Monte Carlo settings
☒	☐	For hierarchical and complex designs, identification of the appropriate level for tests and full reporting of outcomes
☐	☒	Estimates of effect sizes (e.g. Cohen's *d*, Pearson's *r*), indicating how they were calculated

Our web collection on statistics for biologists contains articles on many of the points above.

Software and code

Policy information about availability of computer code

Data collection	Raw and processed GM12878 H3K27me3 and H3K27ac data were obtained from https://www.encodeproject.org/experiments/ENCSR000AKD/ and https://www.encodeproject.org/experiments/ENCSR000AKC/, respectively.<br>Predicted super-silencers and typical silencers in GM12878 are listed in the supplementary tables.
Data analysis	The source code (building the CNN model and predicting enhancers and silencers) is available on GitHub (https://github.com/ncbi/SilencerEnhancerPredict). Data for training (and predicted silencers and enhancers) are available at https://zenodo.org/records/16241561.

For manuscripts utilizing custom algorithms or software that are central to the research but not yet described in published literature, software must be made available to editors and reviewers. We strongly encourage code deposition in a community repository (e.g. GitHub). See the Nature Portfolio guidelines for submitting code & software for further information.

Data

Policy information about availability of data

All manuscripts must include a data availability statement. This statement should provide the following information, where applicable:

- Accession codes, unique identifiers, or web links for publicly available datasets
- A description of any restrictions on data availability
- For clinical datasets or third party data, please ensure that the statement adheres to our policy

H3K27ac and H3K27me2 in primary B cell: https://www.encodeproject.org/biosamples/ENCBS857XIR/

H3K27ac and H3K27me3 data for Ly1 cells: ENCODE EFO:0005907.
H3K27ac and H3K27me3 data for Karpas cells: ENCODE EFO:000719.
H3K27ac and H3K27me3 data for SU-DHL-6 cells: ENCODE EFO:0002357.
H3K27ac and H3K27me3 data for MM.s1 cells: ENCODE EFO:0005724.
H3K27ac and H3K27me3 data for HL-60 cells: ENCODE EFO:0002793.
H3K27ac and H3K27me3 data for Ly3 cells: ENCODE EFO:0006712.
ACAT-STARR-seq activity scores: GEO accession GSE181317.
High-resolution dissection of regulatory activity screening (HiDRA): GEO association GSE104001.
DLBLC data (including SEs, BRD4 ChIP-seq signals and peaks, gene expression profiles): GEO accession GSE45630 and GSE46663.
GM12878 methylation data: GEO accession GSE155791
Cancer SNVs and TLBPs: the ICGC as of 2019.
GM12878 Hi-C contacts and TADs: the Peakachu, https://3dgenome.fsm.northwestern.edu/publications.html

Research involving human participants, their data, or biological material

Policy information about studies with human participants or human data. See also policy information about sex, gender (identity/presentation), and sexual orientation and race, ethnicity and racism.

Reporting on sex and gender	We did not perform sex- and gender-based analysis in the study, because the data were collected using established cell lines.
Reporting on race, ethnicity, or other socially relevant groupings	Race and ethnicity characteristics are not relevant to this study.
Population characteristics	Population characteristics are not relevant to this study.
Recruitment	Recruitment is not relevant to this study.
Ethics oversight	N/A

Note that full information on the approval of the study protocol must also be provided in the manuscript.

Field-specific reporting

Please select the one below that is the best fit for your research. If you are not sure, read the appropriate sections before making your selection.

☒ Life sciences ☐ Behavioural & social sciences ☐ Ecological, evolutionary & environmental sciences

For a reference copy of the document with all sections, see nature.com/documents/nr-reporting-summary-flat.pdf

Life sciences study design

All studies must disclose on these points even when the disclosure is negative.

Sample size	Sample sizes are noted in the figures and results.
Data exclusions	No data were excluded from the study.
Replication	Biological experiments were conducted in two trials, as listed in the supplementary tables. All major findings were consistent between trials.
Randomization	Randomization is not applicable for this study.
Blinding	Blinding is not required for this study.

Reporting for specific materials, systems and methods

We require information from authors about some types of materials, experimental systems and methods used in many studies. Here, indicate whether each material, system or method listed is relevant to your study. If you are not sure if a list item applies to your research, read the appropriate section before selecting a response.

Materials & experimental systems

n/a	Involved in the study
[x]	[ ] Antibodies
[ ]	[x] Eukaryotic cell lines
[x]	[ ] Palaeontology and archaeology
[x]	[ ] Animals and other organisms
[x]	[ ] Clinical data
[x]	[ ] Dual use research of concern
[x]	[ ] Plants

Methods

n/a	Involved in the study
[ ]	[x] ChIP-seq
[x]	[ ] Flow cytometry
[x]	[ ] MRI-based neuroimaging

Eukaryotic cell lines

Policy information about cell lines and Sex and Gender in Research

Cell line source(s)	GM12878. Peripheral blood mononuclear cells (PBMCs) were obtained by density-gradient centrifugation from healthy individuals enrolled under NIH Clinical Center Institutional Review Board-approved protocol 99-GC-0168.
Authentication	GM12878 cells were not authenticated.
Mycoplasma contamination	examined cells were not tested for mycoplasma contamination.
Commonly misidentified lines (See ICLAC register)	No commonly misidentified cell lines were used in the study.

Plants

Seed stocks	N/A
Novel plant genotypes	N/A
Authentication	N/A

ChIP-seq

Data deposition

[x] Confirm that both raw and final processed data have been deposited in a public database such as GEO.

[x] Confirm that you have deposited or provided access to graph files (e.g. BED files) for the called peaks.

Data access links *May remain private before publication.*	Publicly-availible ChIP-seq data have been used in this study. No new ChIP-seq data were generated.
Files in database submission	N/A
Genome browser session (e.g. UCSC)	N/A

Methodology

Replicates	N/A
Sequencing depth	N/A
Antibodies	N/A
Peak calling parameters	N/A
Data quality	N/A
Software	N/A
